# Predictors and pattern of weaning and long-term outcome of patients with prolonged mechanical ventilation at an acute intensive care unit in North India

**DOI:** 10.5935/0103-507X.20170005

**Published:** 2017

**Authors:** Syed Nabeel Muzaffar, Mohan Gurjar, Arvind K. Baronia, Afzal Azim, Prabhakar Mishra, Banani Poddar, Ratender K. Singh

**Affiliations:** 1 Department of Critical Care Medicine, Sanjay Gandhi Postgraduate Institute of Medical Sciences - Lucknow, Índia.; 2 Department of Biostatistics & Health Informatics, Sanjay Gandhi Postgraduate Institute of Medical Sciences - Lucknow, Índia.

**Keywords:** Respiration, artificial, Weaning, Critical illness/mortality

## Abstract

**Objective:**

This study aimed to examine the clinical characteristics, weaning pattern, and outcome of patients requiring prolonged mechanical ventilation in acute intensive care unit settings in a resource-limited country.

**Methods:**

This was a prospective single-center observational study in India, where all adult patients requiring prolonged ventilation were followed for weaning duration and pattern and for survival at both intensive care unit discharge and at 12 months. The definition of prolonged mechanical ventilation used was that of the National Association for Medical Direction of Respiratory Care.

**Results:**

During the one-year period, 49 patients with a mean age of 49.7 years had prolonged ventilation; 63% were male, and 84% had a medical illness. The median APACHE II and SOFA scores on admission were 17 and 9, respectively. The median number of ventilation days was 37. The most common reason for starting ventilation was respiratory failure secondary to sepsis (67%). Weaning was initiated in 39 (79.5%) patients, with success in 34 (87%). The median weaning duration was 14 (9.5 - 19) days, and the median length of intensive care unit stay was 39 (32 - 58.5) days. Duration of vasopressor support and need for hemodialysis were significant independent predictors of unsuccessful ventilator liberation. At the 12-month follow-up, 65% had survived.

**Conclusion:**

In acute intensive care units, more than one-fourth of patients with invasive ventilation required prolonged ventilation. Successful weaning was achieved in two-thirds of patients, and most survived at the 12-month follow-up.

## Abstract (pt)

**Objetivo:**

Examinar as características clínicas, o padrão de desmame e o desfecho de pacientes que necessitaram de ventilação mecânica por tempo prolongado em uma unidade de terapia intensiva em um país com recursos financeiros limitados.

**Métodos:**

Estudo prospectivo observacional em centro único, realizado na Índia, no qual todos os pacientes adultos que necessitaram de ventilação mecânica prolongada foram acompanhados quanto a duração e padrão do desmame, e à sobrevivência, tanto por ocasião da alta da unidade de terapia intensiva quanto após 12 meses. A definição de ventilação mecânica prolongada adotada foi a do consenso da *National Association for Medical Direction of Respiratory Care*.

**Resultados:**

Durante o período de 1 ano, 49 pacientes com média de idade de 49,7 anos receberam ventilação mecânica prolongada; 63% deles eram do sexo masculino e 84% tinham uma enfermidade de natureza clínica. As medianas dos escores APACHE II e SOFA quando da admissão foram, respectivamente, 17 e 9. O tempo mediano de ventilação foi 37 dias. A razão mais comum para início da ventilação foi insuficiência respiratória secundária à sepse (67%). O desmame foi iniciado em 39 (79,5%) pacientes, com sucesso em 34 deles (87%). A duração mediana do desmame foi de 14 (9,5 - 19) dias, e o tempo mediano de permanência na unidade de terapia intensiva foi 39 (32 - 58,5) dias. A duração do suporte com vasopressores e a necessidade de hemodiálise foram preditores independentes significantes de insucesso no desmame. No acompanhamento após 12 meses, 65% dos pacientes sobreviveram.

**Conclusão:**

Mais de um quarto dos pacientes com ventilação invasiva na unidade de terapia intensiva necessitaram de ventilação mecânica prolongada. Os desmames foram bem-sucedido em dois terços dos pacientes, e a maioria deles sobreviveu até o acompanhamento após 12 meses.

## INTRODUCTION

Prolonged mechanical ventilation (MV) is not uncommon in critically ill patients and is defined as when extended periods of MV and other life support therapies are required to support ongoing organ failure.^(1)^ Prolonged MV presents a multitude of problems including the consumption of a substantial amount of intensive care unit (ICU) resources in terms of hospital staff, ICU beds and expenditure and the emotional and financial burden placed on a patient's family.^(2,3)^

Prolonged MV has been defined in the literature as MV for > 24 hours, > 96 hours, 7 days, > 29 days and the need for post-ICU MV support.^(4,5)^ The discrepancy in terminology, admission and discharge criteria and the heterogeneity in population cohorts have led to variation in the epidemiology and outcome of prolonged MV.^(6)^ In the 2005 consensus conference of the National Association for Medical Direction of Respiratory Care (NAMDRC), prolonged MV was defined as requiring MV for ≥ 6 hours/day for more than 21 consecutive days.^(6)^ Using the definition outlined in the NAMDRC consensus conference, single-center studies have shown an incidence of prolonged MV of approximately 3 - 14%.^(7,8)^ Most of the data regarding the epidemiology of prolonged MV have emerged from post-ICU care or specialized weaning units.^(9-11)^ There is a paucity of data from acute ICU facilities. In developing countries, due to the lack of specialized respiratory and weaning units, these patients are continuously managed in acute ICUs.

The aim of this prospective, observational study was to examine the clinical characteristics, weaning pattern, and outcome of patients requiring prolonged mechanical ventilation in acute intensive care unit settings of a resource-limited country using the definition of prolonged mechanical ventilation determined in the NAMDRC consensus conference.

## METHODS

This study was conducted in accordance with the amended Declaration of Helsinki. The Local Institutional Ethics Committee (IEC) approved the protocol; the waiver of consent was granted by the IEC of Sanjay Gandhi Postgraduate Institute of Medical Sciences (IEC code 2014-106-DM-EXP).

This study was a prospective observational study on the clinical characteristics, weaning pattern and long-term outcome of critically ill adult patients requiring prolonged MV in a 12-bed mixed medical and surgical closed ICU of a tertiary care teaching institute in North India. Due to the lack of a specialized weaning and rehabilitation unit, patients were managed in the same acute ICU until they were liberated from MV. Senior registrar and staff nurses were present around the clock, and one physiotherapist was available during the day time.

The total duration of the study was 12 months. All patients > 18 years of age who required invasive MV were followed up and subsequently included in the study if they required invasive MV for ≥ 6 hours/day for > 21 consecutive days. Patients who had known chronic neuromuscular disease, an unclear duration of ventilation pre-ICU admission, or had care withheld or withdrawn during their ICU stay were excluded from this study.

All patients were managed according to the treating physician's decision. The treating physician decided to initiate weaning when it was considered feasible in the course of MV; this decision included daily assessments of readiness to wean from MV, followed by a spontaneous breathing trial (SBT) if the patient was ready. The parameters for assessing readiness to wean from a ventilator in our ICU generally include (but are not limited to) the following criteria: adequate sensorium with intact airway protection or presence of a tracheostomy tube in situ, lack of excess secretions, hemodynamic stability, ratio of partial pressure of arterial oxygen to fraction of inspired oxygen > 200 with ventilator set to deliver positive end-expiratory pressure (PEEP) ≤ 5cmH_2_O, and signs of resolution of the underlying condition for which the patient was ventilated. The parameters for considering SBT and the type and duration of SBT were determined by the treating physician. If SBT failed, MV was re-started. Once the patient was considered ready to wean again, another SBT was performed until the patient was successfully liberated from MV. Some other important used definitions in this study were: weaning success (successful liberation from MV), defined as successful liberation from MV without any requirement of MV (invasive or non-invasive) for at least 48 hours after discontinuation of MV; weaning failure, defined as the resumption of ventilatory support within 48 hours of discontinuation of MV; weaning duration, defined as the number of days between the first SBT to successful liberation from MV.

For the included patients, demographic and clinical characteristics and Acute Physiology and Chronic Health Evaluation (APACHE II) and Sequential Organ Dysfunction Assessment (SOFA) scores at ICU admission were recorded in a structured proforma. During ICU stays, the presence of acute respiratory distress syndrome, septic shock, acute kidney injury, need for hemodialysis and delirium was recorded. Use of neuromuscular blockers, steroids, and vasopressors (≥ 6 hours/day), duration of shock (in days), maximum PEEP requirement (≥ 6 hours/day), duration of MV (days) and total dosage of sedative drugs were also recorded. Acute respiratory distress syndrome, septic shock and delirium were defined according to standard definitions, namely the Berlin definition, Surviving Sepsis Guidelines and Confusion Assessment Method for the Intensive Care Unit (CAM-ICU), respectively.

At first SBT, the type and duration of SBT, need for inotropes, need for hemodialysis, fluid balance in last 3 days (mL), and hemoglobin (g/dL), albumin (g/dL), and phosphate (mg/dL) levels were noted. Any complications after the first SBT (shock, ventilator-associated pneumonia, delirium) were recorded. Any extubation attempts during the ICU stay were also recorded. The date of tracheostomy, of successful liberation from MV, and of decannulation and the duration of weaning (in days) were recorded. The denominator used for successful liberation was the number of patients who received SBT. Length of ICU stay (in days) and mortality at ICU discharge were recorded. In the long-term follow up, survival at 12 months post-inclusion in the study was assessed by telephone.

### Statistical analysis

Continuous variables were expressed as the mean ± standard deviation or the median (inter-quartile range "IQR") depending on the normality of the data. Normality of the data was checked using Shapiro-Wilk's test. Categorical data were expressed as frequencies and percentages. Pearson's chi-square test and Fisher's exact test (univariate analysis) were used to test the associations between dichotomous outcome and each of the individual variables. Univariate binary logistic regression analysis was performed to calculate odds ratios, while multivariate binary logistic regression was used to calculate adjusted odds ratios. Similarly, in the survival analysis, univariate and multivariate analyses were conducted to calculate hazard ratios (HRs) for variables associated with twelve-month mortality by Cox proportional hazards model. For the multivariate analysis in both the binary logistic regression and Cox proportional hazard models, we included variables from the univariate analysis with a p value < 0.05. Kaplan-Meier method with log rank test was used to compare the probability of survival between the groups in the 12-month survival analysis. Statistical significance was established when the p value was less than 0.05. Data were analyzed using Statistical Package for Social Sciences (SPSS), version 22 (IBM, Chicago, USA).

## RESULTS

**Figure 1:**
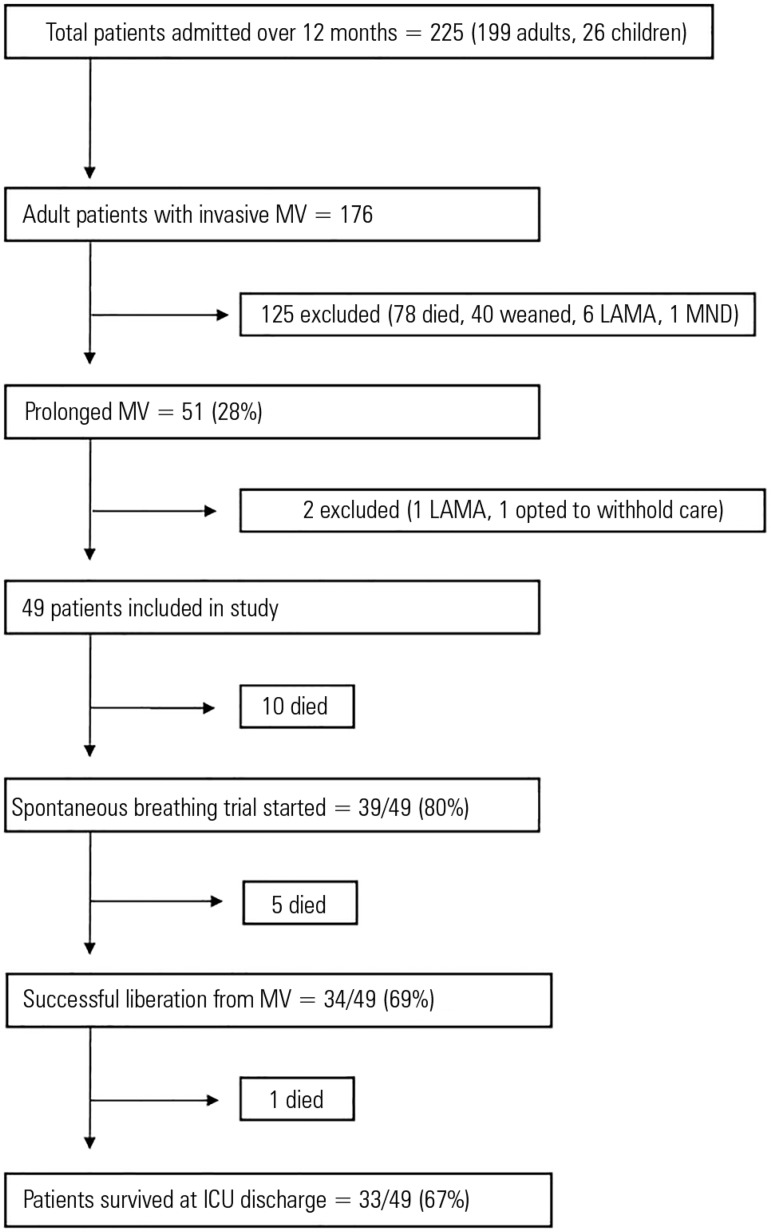
Study flow chart for patients with prolonged mechanical ventilation. MV - mechanical ventilation; LAMA - left against medical advice; MND - motor neuron disease; ICU - intensive care unit.

Over the study period (May 2014 - April 2015), 225 patients were admitted to the ICU (adults: 199 and children: 26). Of these, 176 adult patients required invasive MV, 51 of whom had prolonged MV (28%); 49 patients with prolonged MV were included in this study, and 2 patients were excluded (1 left against medical advice and 1 opted to withhold treatment after prolonged MV) (Figure 1). Of the 49 patients, 21 (43%) were admitted from other hospitals, 20 (41%) were transferred from other units within the hospital, and 8 (16%) were admitted directly from the emergency ward.

At admission, MV had already been initiated in all but 3 patients (who received MV after 24 hours, 72 hours or 12 days of ICU stay). Eight patients had a tracheostomy tube in situ, and the rest had an oral endotracheal tube. The mean age of the included patients was 49.7 years, 63% were male, and 84% had a medical illness. Approximately two-thirds of patients had co-morbidities. Most of them had cardiac co-morbidities (coronary artery disease) (41%) followed by diabetes mellitus (33%), chronic kidney disease (12%), respiratory co-morbidities (chronic obstructive pulmonary disease, asthma, or history of pulmonary tuberculosis) (10%), and both respiratory and cardiac co-morbidities (4%).

The median (IQR) APACHE II and SOFA scores on admission were 17 (14 - 21) and 9 (7 - 12), respectively. The most common reasons for initiating MV were respiratory failure due to severe sepsis/septic shock (67%) and altered sensorium (49%). The median (IQR) MV duration was 37 (28 - 56.5) days, and the median value of maximum PEEP required was 10 (9 - 12).

Neuromuscular blockade (atracurium) was used in 45% and steroids in 63% of patients. Shock was present in 45 (92%) patients, and the mean number of shock days was 13.3 ± 10.4. Forty (82%) patients had acute kidney injury, of whom 23/40 (57.5%) required hemodialysis. Delirium was present in 22 (45%) patients. The median length of ICU stay was 39 (32 - 58.5) days. Overall, 16 (33%) patients had died at ICU discharge (Table 1).

**Table 1:** Characteristics and outcome of patients with prolonged mechanical ventilation

Demographic and clinical characteristics (All patients: N=49)	Mean ± SD	N (%)	Median (25% - 75%)
Age (years)	49.7 ± 16.7		
Male		31 (63)	
Co-morbidities			
Cardiac		20 (41)	
Diabetes mellitus		16 (33)	
Chronic kidney disease		6 (12)	
Hypothyroidism		6 (12)	
Respiratory		5 (10)	
Both respiratory and cardiac		2 (4)	
Medical illness		41 (84)	
SOFA score at admission			9 (7 - 12)
APACHE II score at admission			17 (14 - 21)
Reason to start MV			
Respiratory failure		33 (67)	
Severe sepsis/septic shock		21 (43)	
Pneumonia		21 (43)	
Aspiration		15 (31)	
Trauma		2 (4)	
Postoperative		2 (4)	
Altered sensorium		24 (49)	
ARDS		12 (24)	
Mild		4 (33)	
Moderate		3 (25)	
Severe		5 (42)	
PEEP maximum (cmH20)			10 (9 - 12)
Duration of MV (days)			37 (28 - 56.5)
Presence of shock		45 (92)	
Duration of vasopressors (days)			10 (5 - 20)
Acute kidney injury, n %		40 (82)	
Need for hemodialysis		23 (47)	
Dose of sedative drugs per patient during ICU stay			
Midazolam (mg)	622.2 ± 580.2	47 (96)	
Fentanyl (µg)	28.311.8 ± 26.326.3	46 (94)	
Dexmedetomidine (µg)	742.1 ± 1.853.3	19 (39)	
Propofol (mg)	177.3 ± 341.9	23 (47)	
Presence of delirium		22 (45)	
Use of neuromuscular blockade		22 (45)	
Use of steroid		31 (63)	
**At first spontaneous breathing trial (N=39; 80%)**	**Mean ± SD**	**N (%)**	**Median** **(25% - 75%)**
Patients with oral endotracheal tube		11 (28)	
On hemodialysis		7 (18)	
Need for dobutamine support		3 (8)	
Fluid balance in last 3 days (mL)			1.000 (-400 - 2.000)
Negative balance		12 (31)	
Positive balance (< 500 mL)		5 (13)	
Positive balance (500 - 1000 mL)		4 (10)	
Positive balance (> 1000 mL)		18 (46)	
Laboratory values			
Hemoglobin (g/dL)	8.8 ± 1.6		
Albumin (g/dL)	2.8 ± 1.8		
Phosphate (mg/dL)	4.4 ± 1.9		
Potassium (mEq/L)	3.8 ± 0.9		
Extubation attempts		9 (23)	
Successful extubation		1/9	
Time to tracheostomy from MV (days)			11 (8 - 16.5)
**Outcome of patients who received spontaneous breathing trial**	**Mean ± SD**	**N (%)**	**Median** **(25% - 75%)**
Successful weaning achieved		34 (87)	
Length of stay in ICU (days)			39 (32 - 58.5)
ICU mortality		16 (33)	
12-month mortality		17 (34)	

SD - standard deviation; SOFA - Sequential Organ Dysfunction Assessment; APACHE II - Acute Physiology and Chronic Health Evaluation II; MV - mechanical ventilation; ARDS - acute respiratory distress syndrome; PEEP - positive end-expiratory pressure; AKI - acute kidney injury; ICU - intensive care unit.

Overall, SBT was initiated in 39 (80%) patients. In 15 patients, the first SBT was initiated after 21 days of consecutive MV. T-piece trial was the only SBT method used by the treating physicians. Prior to the first SBT, 11 patients had an oral endotracheal tube. Extubation attempts were undertaken in 9 patients but were successful in only 1. The remaining patients received a tracheostomy (Table 1).

Among all included patients with prolonged MV (49 patients), 34 (69%) were successfully liberated from MV. Based on the recent classification of weaning, all had prolonged weaning. The median (IQR) duration between first SBT to successful liberation from MV was 14 (9.5 - 19) days. After initiating SBT, 23 (68%) patients suffered from ventilator-associated pneumonia, 18 (53%) had shock, and 13 (38%) had delirium. After successful liberation, 9 patients (26%) required re-institution of MV during their ICU stay; one died, while the others were liberated successfully again after subsequent SBT trials. At ICU discharge, no patient was ventilator dependent, while 6 (18%) patients were discharged with a tracheostomy tube in situ due to their inability to protect their airway. The median (IQR) length of ICU stay of these patients was 41 (33.7 - 72.75) days (Table 2). Of the 33 patients who were alive at ICU discharge, 30 were directly discharged to home.

**Table 2:** Weaning pattern and outcome of patients with successful liberation from mechanical ventilation (N = 34)

**Pattern of weaning**	
Total duration from first spontaneous breathing trial to successful liberation from MV (days)	14 (9.5 - 19)
**New problems after first spontaneous breathing trial**	
Ventilator-associated pneumonia	23 (68)
Shock	18 (53)
Delirium	13 (38)
Mechanical ventilation after successful liberation	9 (26)
**Outcome of patients**	
Length of stay in ICU	41 (33.7 - 62.75)
ICU mortality	1 (3)
12-month mortality	2 (6)

MV - mechanical ventilation; ICU - intensive care unit. The results are expressed as the median (25% - 75%) and number (%).

**Figure 2:**
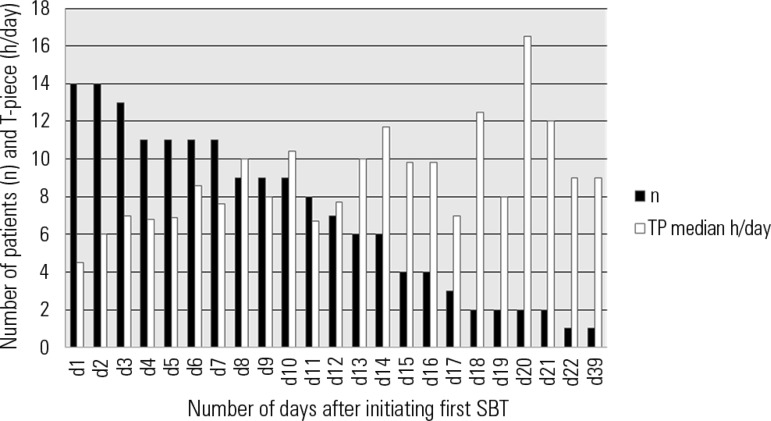
Mean duration of T-piece (hours/day) until successful liberation among patients with first spontaneous breathing trial provided after 21 days of mechanical ventilation (N=14). SBT - spontaneous breathing trial; D - day.

The pattern of weaning duration of patients (i.e., median hours of T-piece per day from their first SBT day until successful liberation from MV) who received their first SBT after 21 consecutive days of MV is shown in figure 2. Among this group, more than half of the patients were successfully liberated within 2 weeks after initiating SBT.

Table 3 shows the univariate and multivariate binary logistic regression analysis of the factors associated with successful liberation from MV. The duration of vasopressor support and the need for hemodialysis were found to be significant independent predictors and to be negatively associated with successful liberation in both the univariate and multivariate analysis (p < 0.05).

**Table 3:** Univariate and multivariate predictors of successful liberation from prolonged mechanical ventilation

Variables	Binary logistic regression analysis			
	Univariate		Multivariate	
	OR (95%CI)	p value	AOR (95%CI)	p value
Age (years)				
< 40	1.57 (0.49 - 5.618)	0.540		
40 - 70	0.50 (0.14 - 1.77)	0.360		
Sex				
Male (versus female)	0.51 (0.13 - 1.97)	0.517		
Co-morbidities				
Diabetes mellitus	0.54 (0.14 - 1.95)	0.497		
Chronic obstructive pulmonary disease	3.44 (0.16 - 70.90)	0.540		
Cardiomyopathy	1.35 (0.12 - 14.20)	1.000		
Chronic kidney disease	0.17 (0.02 - 1.07)	0.060		
Medical illness	0.30 (0.03 - 2.76)	0.410		
SOFA (at admission)	-0.07	0.450		
APACHE II (at admission)	0.03	0.640		
Reason for initiation of MV				
Low Glasgow Coma Scale score	0.77 (0.23 - 2.62)	0.760		
Pneumonia	0.80 (0.23 - 2.71)	0.760		
Aspiration	0.35 (0.09 - 1.27)	0.170		
Sepsis	1.04 (0.28 - 3.80)	1.000		
ARDS	1.44 (0.32 - 6.30)	0.730		
Mild	1.35 (0.12 - 14.20)	1.000		
Moderate	3.44 (0.16 - 70.98)	0.540		
Severe	0.62 (0.09 - 4.21)	0.630		
Clinical course during ICU stay				
PEEP (Maximum)	0.12	0.460		
Duration of vasopressor support (days)	0.12	0.004	0.11	0.042
Duration of MV (days)	0.01	0.380		
Acute kidney injury	0.23 (0.02 - 2.05)	0.240		
Need for hemodialysis	0.06 (0.01 - 0.33)	0.0004	0.67 (0.07 - 0.92)	0.018
Use of sedative drugs	0.41 (0.01 - 9.27)	1.000		
Presence of delirium	1.33 (0.38 - 4.57)	0.750		
Neuromuscular blockade	0.61 (0.18 - 2.08)	0.530		

OR - odds ratio; 95%CI - 95% confidence interval; AOR - adjusted odds ratio; SOFA - Sequential Organ Dysfunction Assessment; APACHE II - Acute Physiology and Chronic Health Evaluation II; MV - mechanical ventilation; ARDS - acute respiratory distress syndrome; ICU - intensive care unit; PEEP - positive end-expiratory pressure. Outcome (success or failure): for categorical variables, odds ratio (95% confidence interval) [p value], for continuous variables. Beta coefficient [p value]. Factors with a p value < 0.05 in the univariate analysis were included in the multivariate analysis. Statistical significance was indicated when p value < 0.05.

Table 4 shows the univariate and multivariate Cox proportional hazards analysis conducted to identify the independent predictors of mortality at 12 months from ICU admission. The results show that in the multivariate analysis, the use of neuromuscular blockade was associated with increased mortality (HR = 3.78; p = 0.033) while the duration of MV (days) was associated with decreased mortality (ß = -0.02; p = 0.047) (Table 4).

**Table 4:** Univariate and multivariate predictors of one-year mortality in patients with prolonged mechanical ventilation

Variables	Cox proportional hazards analysis			
	Univariate		Multivariate	
	HR (95%CI)/Beta	p value	HR (95%CI)/Beta	p value
Age (years)	0.01	0.460		
Gender (male versus female)	0.78 (0.28 - 2.17)	0.630		
Co-morbidities				
Diabetes mellitus	0.65 (0.252 - 1.676)	0.370		
Coronary artery disease	0.75 (0.246 - 2.279)	0.610		
Cardiomyopathy	1.77 (0.235 - 13.27)	0.580		
Chronic kidney disease	0.44 (0.14 - 1.345)	0.150		
Medical illness	1.64 (0.359 - 7.521)	0.520		
SOFA (at admission)	-0.08	0.397		
APACHE II (at admission)	-0.06	0.118		
Reason for initiation of MV				
Low Glasgow Coma Scale score	0.79 (0.31 - 1.99)	0.610		
Pneumonia	0.98 (3.89 - 2.49)	0.970		
Postoperative respiratory failure	0.55 (0.07 - 4.18)	0.570		
Sepsis	1.16(0.43 - 3.08)	0.770		
ARDS	0.66 (0.235 - 1.858)	0.43		
Mild	0.69 (0.152 - 3.18)	0.64		
Moderate	0.53 (0.065 - 4.302)	0.55		
Severe	0.36 (0.07 - 1.731)	0.20		
Clinical course during ICU stay				
PEEP (Maximum)	-0.03	0.830		
Duration of shock (days)	0.01	0.610		
Acute kidney injury	0.45 (0.10 - 1.96)	0.290		
Presence of delirium	1.04 (0.41 - 2.64)	0.930		
Use of neuromuscular blockade	0.45 (0.17 - 1.15)	0.003	3.78(1.73 - 13.07)	0.033
Duration of MV (days)	-0.09	0.003	-0.02	0.047

HR - hazard ratio; 95%CI - 95% confidence interval; SOFA - Sequential Organ Dysfunction Assessment; APACHE II - Acute Physiology and Chronic Health Evaluation II; MV - mechanical ventilation; ARDS - acute respiratory distress syndrome; ICU - intensive care unit; PEEP - positive end-expiratory pressure. Outcome (death). For categorical variables, hazard ratio, 95% confidence interval (p value); for continuous variables, beta coefficient (p value). Factors with a p value < 0.05 in the univariate analysis were included in the multivariate analysis. Findings were considered statistically significant at p value < 0.05.

**Figure 3:**
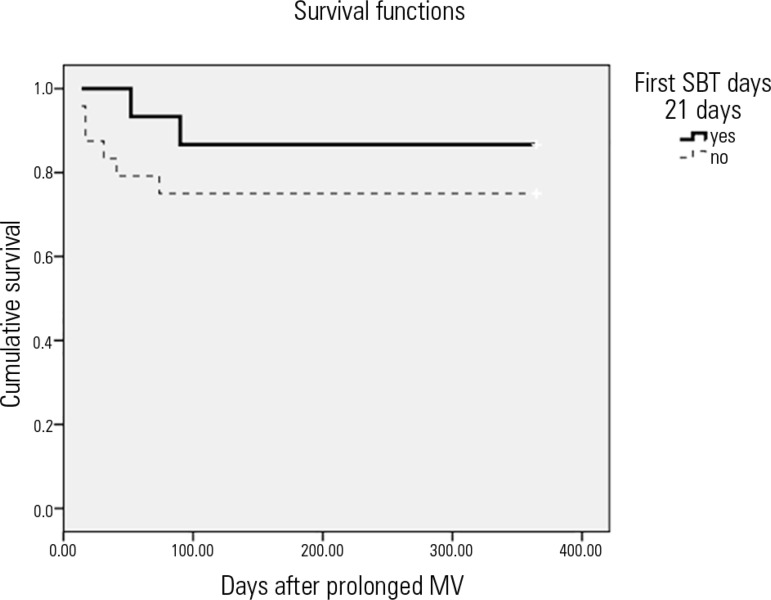
One-year Kaplan-Meier survival curve for patients with prolonged mechanical ventilation who received a spontaneous breathing trial. Yes: first SBT initiated after 21 consecutive days of MV; No: first SBT before 21 days of MV; p = 0.31. SBT - spontaneous breathing trial; MV - mechanical ventilation.

In the long-term (12 months) follow-up of patients with prolonged MV, Kaplan-Meier analysis was used to compare the probability of survival between the two groups of patients who received SBT. The results indicated that there was no significant difference in the probability of survival between patients who received their first SBT after 21 days of MV compared to those who received their first SBT before 21 days of MV (Figure 3; p = 0.31).

## DISCUSSION

The incidence of prolonged MV in acute ICUs has been reported to range from 3 - 14%, mainly depending on the definition of prolonged MV used. Based on the definition outlined in the NAMDRC, we found that the incidence of prolonged MV was as high as 28% in our study. This rate is higher compared to studies in other countries (where prolonged MV was defined as MV > 21 days) such as Argentina (14%), the UK (6%) and Brazil (10%).^(3,12-14)^ Possible reasons for the higher incidence in our study were as follows: as the study was conducted in a referral center and as there was a lack of sufficient ICU beds in the region, we received sicker patients in our ICU (more than 90% had shock); additionally, approximately two-thirds of patients had ventilator-associated pneumonia after their first SBT. The lack of weaning units may be another reason for the high incidence of prolonged MV. Thus, all patients were admitted and managed in the same acute ICU until discharge (unlike other ICUs).

The definition of successful liberation from MV used in previous studies has varied (e.g., no requirement of MV after discontinuation for 48 hours, 7 days, 14 days or up to 6 months-1 year).^(6,15)^ We defined successful liberation as a lack of need for MV for up to 48 hours after discontinuation of MV. Using this definition, two-thirds of patients were successfully liberated from MV in our study. Earlier studies have also shown that up to 38-68% of patients are weaned off of MV.^(9,10,15,16)^ Similarly, 50% of patients were found to be successfully liberated from MV in a systematic review and meta-analysis conducted by Damuth et al., which included 124 studies on prolonged MV patients (MV > 14 days) admitted to ventilator units or with a tracheostomy tube in situ for acute respiratory failure.^(14)^

The current literature does not provide a standard weaning protocol or strategy for weaning these patients.^(17)^ The weaning strategy for MV patients differs from that for non-prolonged MV patients.^(18)^ Recently, Jubran et al. compared two weaning strategies (pressure support versus tracheostomy collar) in patients requiring prolonged MV.^(19)^ However, there was no strong evidence in favor of any specific weaning strategy. Similar to other studies, daily SBTs were used in our study and constituted daily T-piece trials of progressively increasing duration.^(12,15,20)^ In our study, more than half of the patients were weaned off MV within the first 2 weeks of SBT, as in a study by Bigatello et al.^(15)^ It is difficult to predict how many patients can be successfully weaned from MV.^(21-23)^ In our study, none of the factors were independently in favor of successful liberation from MV. However, the duration of shock and need for hemodialysis were independently predicted to decrease the chance of successful liberation from MV. This observation is similar to that made by Estenssoro et al.^(13)^

Most of the data regarding outcomes have originated from long-term acute care units or specialized weaning units.^(24-27)^ In a study from Brazil by Ibrahim et al., in which a noninvasive portable ventilator was used in patients following tracheostomy (n = 26) with prolonged MV and with prolonged weaning to facilitate discharge, the mortality rates were 23% and 46% at the time of ICU and hospital discharge, respectively.^(28)^ In a meta-analysis and systematic review by Damuth et al., the pooled mortality at hospital discharge was 29%.^(14)^ Similarly, in our study, 16 (33%) patients did not survive at ICU discharge.

In the long-term follow up, of all patients discharged alive from the ICU, only 1 died (i.e., the 12-month mortality post-study inclusion was 35%). However, in the meta-analysis and systematic review by Damuth et al., the pooled mortality at 1 year was as high as 59%.^(14)^ This discrepancy in our study regarding long-term outcomes can be explained by the fact that patients in our ICU were stable enough to be discharged directly to home. Thus, their long-term survival was better. Unlike studies from other acute ICUs included in the meta-analysis, transfer to rehabilitation centers, to long-term or specialized weaning units or to other acute ICUs was not required in our patients.

A limitation of our study is that it was a single-center study and that only a small number of patients were studied over the 1-year period. Moreover, no comparisons were made with non-prolonged MV patients.

On the other hand, the strengths of the study are its prospective design and the fact that it is one of the few studies conducted in a resource-limited setting.^(12-14,28-30)^

## CONCLUSION

In our study, the incidence of prolonged mechanical ventilation was as high as 27% of patients requiring invasive mechanical ventilation, and successful weaning was achieved in two-thirds of these patients. Duration of shock (OR, 0.097; p = 0.04) was found to be an independent predictor of failed liberation from MV in the multivariate analysis. The median (IQR) weaning duration was 14 (9.5 - 19) days. Of the patients who received their first spontaneous breathing trial after 21 consecutive days of mechanical ventilation, more than half were successfully liberated within 2 weeks after initiating the SBT. The majority of patients who were alive at ICU discharge were discharged directly to home. In addition, at the 12-month follow-up, all but one had survived.

This study highlights that in resource-limited countries, where there is an acute shortage of intensive care beds, many prolonged ventilated patients continue to be managed in acute care settings. The findings also highlight the urgent need for specialized weaning units to be created by policy makers.

### Contribution of authors

As principal investigator, Mohan Gurjar had full access to all the study data and assumes responsibility for the integrity of the data and the accuracy of the analysis. Syed Nabeel Muzaffar, Mohan Gurjar, Arvind K. Baronia and Afzal Azim contributed to the study's conception, design, and interpretation. Syed Nabeel Muzaffar and Mohan Gurjar were responsible for searching the literature; Syed Nabeel Muzaffar, Mohan Gurjar, Arvind K. Baronia and Prabhakar Mishra were responsible for screening abstracts, selecting manuscripts for full-text review, and performing the analysis; and Syed Nabeel Muzaffar, Mohan Gurjar, Arvind K. Baronia, Afzal Azim, Prabhakar Mishra, Banani Poddar and Ratender K. Singh assisted in the successive revisions of the final manuscript and read and approved the final manuscript. All authors read and approved the final manuscript.
